# Enhancing droplet deposition through *in-situ* precipitation

**DOI:** 10.1038/ncomms12560

**Published:** 2016-08-30

**Authors:** Maher Damak, Seyed Reza Mahmoudi, Md Nasim Hyder, Kripa K. Varanasi

**Affiliations:** 1 Department of Mechanical Engineering, Massachusetts Institute of Technology, 77 Massachusetts avenue, Cambridge, Massachusetts 02139, USA

## Abstract

Retention of agricultural sprays on plant surfaces is an important challenge. Bouncing of sprayed pesticide droplets from leaves is a major source of soil and groundwater pollution and pesticide overuse. Here we report a method to increase droplet deposition through *in-situ* formation of hydrophilic surface defects that can arrest droplets during impact. Defects are created by simultaneously spraying oppositely charged polyelectrolytes that induce surface precipitation when two droplets come into contact. Using high-speed imaging, we study the coupled dynamics of drop impact and surface precipitate formation. We develop a physical model to estimate the energy dissipation by the defects and predict the transition from bouncing to sticking. We demonstrate macroscopic enhancements in spray retention and surface coverage for natural and synthetic non-wetting surfaces and provide insights into designing effective agricultural sprays.

## Abstract (web summary)

The extensive use of pesticides in agriculture calls for efficient spraying techniques to reduce pollution of soils and groundwater by toxic chemicals. Damak *et al*. simultaneously spray liquids containing oppositely charged polyelectrolytes that form defects, pinning droplets on targeted surfaces.

## Graphical abstract

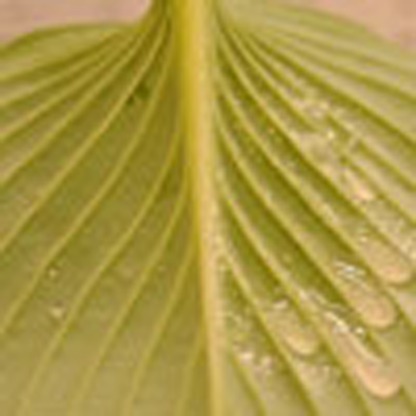

Sprays are used in a wide range of applications, including agriculture, paints, coatings, cosmetics and medicine. In agriculture, sprays are the most common means to deliver pesticides, some nutrients and other chemicals to plants^1^,^2^,^3^. Some of these chemicals, especially pesticides, are toxic, and there is an increasing demand to reduce their use^4^. A study found that pesticides could be detected 90% of the time in agricultural streams, 50% in shallow wells and 33% in major deep aquifers across the USA^5^. Therefore, there is a significant need to eliminate or substantially reduce the sources of deposition inefficiencies in sprays. One of the most important inefficiencies arises from the low retention of sprayed liquids on plant surfaces due to their hydrophobic/superhydrophobic properties—droplets from sprays impacting plant surfaces can bounce or roll off plant surfaces. Such plants are common, and they usually get their non-wetting properties from the presence of waxy features on their surface^6^,^7^,^8^. As a result, most of the liquid from agricultural sprays ends up in the soil, polluting it and contaminating groundwater^9^,^10^. Similarly, low retention of sprayed water leads to significant water consumption in frost protection of plants^3^.

The impact of a liquid droplet on a solid surface has been extensively studied^11^,^12^,^13^,^14^,^15^,^16^,^17^,^18^,^19^,^20^,^21^,^22^,^23^. It is known that for a hydrophobic surface, at moderate impact velocities, the droplet undergoes an expansion phase, driven by inertial forces, followed by a retraction phase, driven by surface tension, that may lead to a bounce-off. The outcome of the impact, whether the droplet adheres or bounces off, depends on multiple factors related to the surface and sprayed liquid. The relevant surface properties are surface energy and surface texture and the drop parameters are surface tension, viscosity and size. Current approaches to improve droplet deposition rely on modifying the fluid properties in a way that enhances retention^6^,^24^,^25^.

One common approach to improve drop retention is to add surfactants to the sprayed liquid in order to reduce the surface tension and promote spreading of droplets on the surface^26^. However, even if surfactants enhance spreading in the static case, their effect in a dynamic impact process is more complex. Surfactant molecules must diffuse from the bulk to the newly formed interfaces, as the drop undergoes dramatic changes in shape. If the timescale of this diffusion is higher than the contact time of the droplet with the solid—that is, the time a droplet spends on the surface before bouncing^18^,^27^, these surfactants will have a smaller effect. Therefore, the appropriate parameter to study is the dynamic surface tension, and, considering these dynamic effects, the role of surfactants in drop retention has been shown to be less pronounced than anticipated^28^,^29^. Moreover, adding surfactants leads to smaller droplets in sprays, which aggravate other problems such as wind drift and evaporation^30^.

Another recent approach is based on modifying the rheological properties of the fluid by adding small amounts of a polymer additive to the sprayed solution^25^,^31^. It has been shown that these dilute polymer solutions delay the retraction phase and lead to the deposition of the droplet. For small amounts of polymer, the viscosity and surface tension of the solution remain approximately the same as water. It was suggested that the observed retention arises from non-Newtonian properties, particularly the extensional viscosity of the polymer solution *η*_e_. Stretching the fluid during expansion and retraction unfolds and deforms high molecular weight polymers leading to significant energy dissipation that can prevent droplet rebound. Improvements in deposition on different surfaces have been reported. However, the underlying physical mechanism is still being investigated^32^,^33^,^34^.

Here we propose an alternate approach to enhance drop retention by altering the target surface properties *in-situ* during the spray process by forming sparse hydrophilic defects onto the substrate. These defects would act to pin the contact line of impinging droplets and suppress bouncing^35^. Inspired by the layer-by-layer deposition techniques^36^,^37^ (though this is a different concept), we create these pinning defects by adding small quantities of oppositely charged polyelectrolytes to separate solutions and spraying them simultaneously using two nozzles, as shown schematically in Fig. 1a. When droplets containing oppositely charged polyelectrolytes come into contact, a precipitation reaction occurs and results in the formation of hydrophilic surface defects that pin the drop to the surface and enhance retention (Fig. 1b). After demonstrating the concept with spray experiments, we study the possible interactions between droplets of the oppositely charged polyelectrolyte solutions by performing individual drop-on-drop experiments and observing the deposited precipitate on the surface through microscopy. We then derive a criterion predicting the transition between bouncing and sticking for two impacting droplets and define a characteristic non-dimensional number for this problem. We finally translate these individual droplet results to sprays and show that simultaneous spraying of polyelectrolyte solutions on synthetic and natural superhydrophobic surfaces leads to a substantial increase in the deposition and retention of the liquid.

## Results

### Spray experiments

To study the effect of precipitation on drop impacts, we used two polyelectrolyte molecules. Linear polyethyleneimine was the positively charged polyelectrolyte, with NH_2_^+^ groups in solution, while polyacrylic acid was the negatively charged polyelectrolyte with COO^−^ groups in solution. The molecular weight of both polyelectrolytes was ∼20,000 g mol^−1^. These polyelectrolytes were dissolved in water at different concentrations. Separately, the polyelectrolyte solutions are very dilute and have physicochemical properties that are close to pure water. In particular, surface tension of all the used solutions was measured to be within 13% of that of water (Supplementary Fig. 1). The electrical interactions between ions in solution depend on their zeta potential, which is a measure of the electrical potential difference between the layer of fluid attached to a molecule or particle and the bulk of the solution. The zeta potential of polyelectrolytes depends on the pH of the solution, and, for our experiments, we chose a pH ∼4.5 for which both potentials are sufficiently high to have a substantial interaction between the two polyelectrolytes^38^,^39^. At this pH ∼4.5, we observed that mixing solutions of these two polyelectrolytes resulted in spontaneous formation of insoluble precipitates in the solution^40^,^41^. The precipitates were observed as whitish residues, and they were formed both in the bulk of the solution and on the surface on which the liquid was deposited. We observed that the surface precipitates are hydrophilic and can strongly pin impinging droplets during impact.

**Figure 1:**
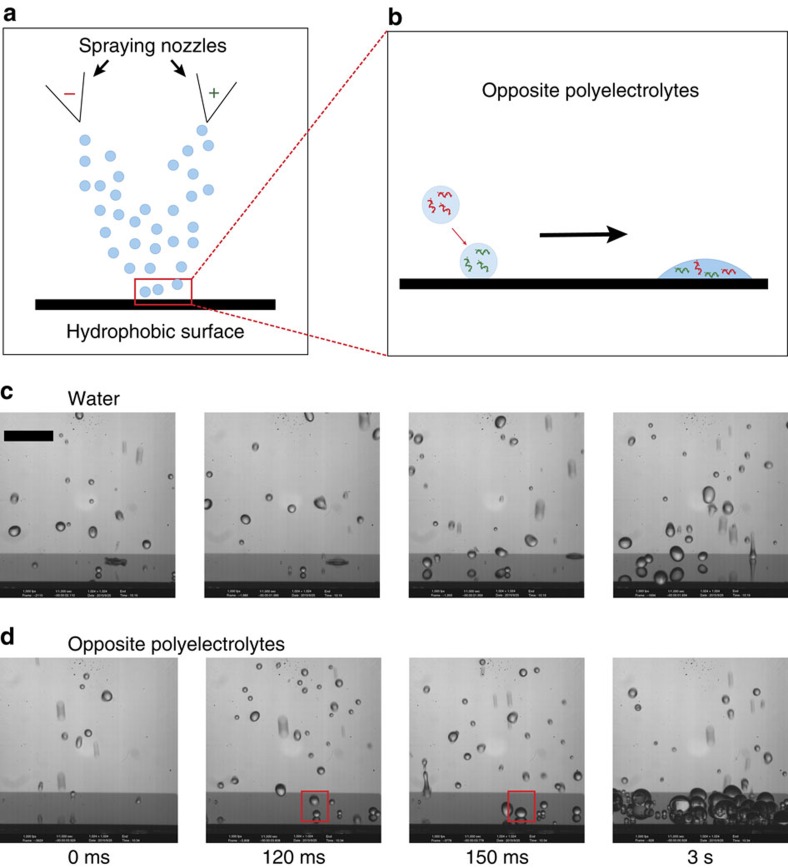
Simultaneous spraying of opposite polyelectrolytes. (**a**) Schematic of experimental set-up for simultaneous spraying of opposite polyelectrolytes. (**b**) Expected behaviour for the impact of a droplet with one polyelectrolyte polarity on a droplet with an oppositely charged polyelectrolyte. The coalesced drop sticks to the surface. (**c**,**d**) Snapshots of simultaneous spraying on a superhydrophobic surface. Sprays with very low droplet density were used to enhance visualization and slow down the process. In the first row, the two sprayers are spraying water and the surface remains dry. Almost all droplets bounce off. Some small droplets are deposited but they are cleared as soon as another droplet impacts them (see Supplementary Movie 1). In the second row, opposite polyelectrolytes are sprayed. Individual droplets hitting the surface still bounce off. After 120 ms of spraying, the first event of a droplet of one polyelectrolyte hitting a droplet containing the opposite polyelectrolyte occurs. The coalesced drop sticks to the surface. Subsequent drops that hit this droplet also coalesce on it. Similar events happen all over the surface. Many droplets can be seen on the surface after 3 s of spraying (see Supplementary Movie 2). Scale bar, 1 cm.

A silicon nanograss surface composed of random features of typical size and spacing around 200 nm and coated with a hydrophobic modifier was used as a model superhydrophobic surface in this study. For this surface, we measured a contact angle of 165° and a contact angle hysteresis smaller than 5° (ref. 42). Simultaneous spraying of water droplets with and without polyelectrolytes was performed on these surfaces and the interaction was captured using a high-speed camera (Fig. 1c,d Supplementary Movies 1 and 2). Sparse sprays were used for better visualization as they slowed down the rate of defect formation. When pure water droplets are sprayed, they bounce off the surface as expected and the surface remains clear as shown in Fig. 1c. Some small droplets may stick to the surface, but as soon as they are impacted by another impinging droplet, they bounce off. In contrast, spraying water droplets containing oppositely charged polyelectrolytes increases liquid retention as shown in Fig. 1d. Close inspection of the spray (Supplementary Movie 2) reveals that not all droplets impacting the surface lead to retention. When single droplets impact the surface they bounce off similar to pure water droplets. Further experiments with impacts of single droplets of either polyelectrolyte show that they bounce off (Supplementary Fig. 2), and spraying only one of the polyelectrolyte solutions results in no retention. Therefore, the retention in simultaneous spraying must come from the interaction between at least two droplets. Indeed, when multiple droplets collide on the surface, the coalesced drop is arrested. The drop is then anchored at the surface and does not detach even when additional droplets impinge on it. The anchored drop continues to grow due to the coalescence of subsequent impacting droplets. As time progresses, the surface is covered with many anchored drops that continue to grow and, after ∼3 seconds of spraying, much of the surface is covered with liquid. We notice that the most frequent case for two-droplet interactions is when small single droplets stay on the surface upon impact and are subsequently impacted by another impinging drop. Thus, we choose drop-on-drop impact experiments as our model experiments.

### Drop-on-drop impacts

**Figure 2:**
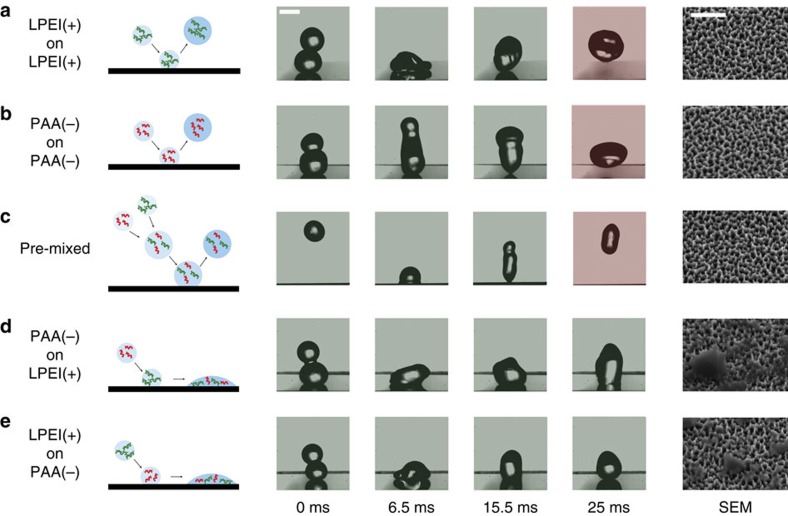
Possible droplet interactions in simultaneous spraying of opposite polyelectrolytes. The first column contains schematics of the five possible scenarios. The next columns are snapshots of individual drop impacts for each of the previous scenarios. The rightmost column contains SEM images of the surface after the impacts. The images were taken at a tilt angle of 30°. Only the last two cases result in arresting the droplet and leaving a residue on the surface. (**a**) A 2 mm droplet of linear polyethyleneimine (LPEI) is deposited on the superhydrophobic surface and another LPEI droplet impacts it vertically. Snapshots of the impact show a similar behaviour to typical single droplet impacts: Upon coalescence the merged droplet expands then retracts and eventually bounces off the surface. The process lasts around 20ms, which is comparable to the contact time of single impinging droplets of similar radius. The SEM image shows the features of the surface, uncovered, as they were before the impact. (**b**) The polyacrylic acid (PAA) on PAA impact exhibits a similar behaviour and results in an unspoiled surface as well. (**c**) LPEI and PAA solutions are premixed, forming a bulk precipitate. The impact of a droplet of this mixture on a superhydrophobic surface results in bouncing, with no satellite droplet left behind. SEM images show that nothing is deposited on the surface. The precipitates seem to remain in the bulk and not act as a pinning site on the surface. (**d**) When a PAA drop impacts an LPEI drop, after a similar expansion phase, the retraction phase ends with an arrested droplet. The SEM image shows the deposition of a residue on the surface, formed by the precipitation of the opposite polyelectrolytes when the two droplets merged on the surface. For complete movie, see Supplementary Movie 3. (**e**) LPEI on PAA is similar and leads to the droplet sticking on the surface. Scale bar, 2 mm (snapshots). Scale bar, 2μm (SEM).

In Fig. 2, we present the five possible cases of two-droplet interactions: A positive droplet (linear polyethyleneimine) impinging on another positive one, a negative droplet (polyacrylic acid) impinging on a negative one, a positive and negative droplet colliding and coalescing in midair and then impinging on the surface, a positive droplet impinging on a negative one and a negative droplet impinging on a positive one. For each of these cases, the drop-on-drop impact experiment is filmed with a high-speed camera at 10,000 frames per second and the impact location of the dried surface is imaged using a scanning electron microscope (SEM).

The results in Fig. 2 show that the impacts involving two droplets containing the same polyelectrolyte result in bouncing in a sequence of spreading and retraction phases that is similar to the impact of a single droplet (Fig. 2a,b). The measured contact time is also close to that of a single droplet (within 15%), and no deposits can be seen in the SEM images. Forming the precipitates beforehand by premixing positive and negative polyelectrolytes before impact does not cause droplets to stick to the surface. None of the bulk precipitates remain on the surface as shown in Fig. 2c. Thus, spraying a solution containing pre-formed precipitates does not increase the liquid retention. The same bouncing behaviour is observed when other hydrophilic particles are added to the droplet. As shown in Supplementary Fig. 3, droplets containing 3 μm silica particles bounce off superhydrophobic surfaces without leaving any particle residue. However, drop-on-drop impacts of droplets with oppositely charged polyelectrolytes lead to droplets sticking to the surface. The droplets are arrested by the pinning sites that are formed due to the *in-situ* precipitation of oppositely charged polyelectrolytes on the surface (Fig. 2d,e).

Studying these different cases confirms that the *in-situ* formation of surface precipitates is the key to enhanced retention and explains what happens when oppositely-charged polyelectrolyte solutions are sprayed: Sufficiently small droplets of one solution may stick to the surface when pinning forces due to the natural hysteresis of the surface overcome inertial forces. Upon impact with another incoming droplet, the coalesced drop will only stick if the two droplets contain oppositely charged polyelectrolytes and can react to form additional surface pinning sites that arrest the coalesced drop.

## Discussion

**Figure 3:**
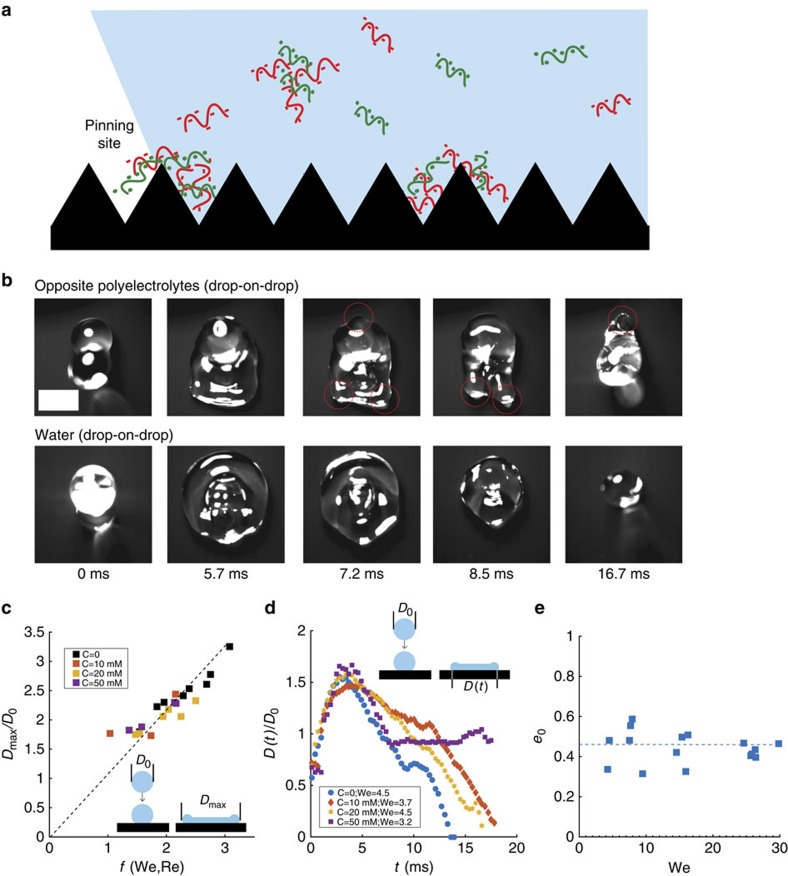
Defects formation and drop-on-drop impact dynamics. (**a**) Schematic of the formation of precipitates in the liquid and the role of surface precipitates in pinning the receding contact line of a droplet. (**b**) Top view snapshots of drop-on-drop impacts. Water droplets exhibit an axisymmetric uniform retraction, while, for polyelectrolytes, retraction is asymmetric and comprises sharp edges, which are the signature of pinning locations (red circles). For complete movie, see Supplementary Movie 4. Scale bar, 2 mm. (**c**) Normalized maximum diameter as a function of the correlation function *f*(We, Re). The Weber number spanned an order of magnitude (4–40) and was varied by using two droplet sizes (1.1 and 1.9 mm radius) and four impact velocities for each. (**d**) Normalized contact length as a function of time for four scenarios. All droplets expand, reach a maximum diameter then retract. Bouncing occurs in three cases where the spreading coefficient goes to zero, while sticking occurs in the case with the highest polyelectrolyte concentration C. (**e**) Restitution coefficient as a function of the impact Weber number for water. The Weber number was varied by changing the droplet size and the impact velocity. In all drop-on-drop experiments, both drops had the same size.

When two droplets containing oppositely charged polyelectrolytes coalesce, there are oppositely charged macromolecules in solution. When these molecules are in close proximity such that the electrical charges are not completely screened, they are attracted to each other and subsequently attract other molecules to form precipitates, as shown in Fig. 3a. Molecules that are close to the surface may be attracted to the surface^43^ and serve as a nucleation site for a surface precipitate. These precipitates act as defects and pin the contact line of the retracting droplet. This pinning dissipates energy and can prevent the droplet from bouncing. As shown in the top view images in Fig. 3b, the retraction front in the case of water droplets is predominantly axisymmetric due to minimal pinning. However, for positive on negative polyelectrolyte drop impact the retracting front is composed of multiple sharp corners and filaments, indicating contact line pinning (see Supplementary Movie 4).

To further characterize these drop-on-drop impacts, we first measure the normalized maximum diameter, defined as the diameter of the coalesced droplet when it reaches its maximal expansion divided by the initial diameter of one droplet *D*_0_. Figure 3c shows the normalized maximum diameter for various drop sizes, impact velocities, and polyelectrolyte concentrations. We find that for our experimental conditions (4<We<40, 500<Re<5000), the normalized maximum diameter scales as 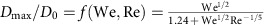, where We is the Weber number and Re is the Reynolds number, as was shown in previous studies for single drop impacts^44^,^45^. The results are identical for the cases with and without polyelectrolytes. Figure 3d shows the time evolution of the normalized contact length *D(t)/D*_0_ for different polyelectrolyte concentrations. We observe that, similar to the normalized maximum diameter, the expansion time, defined as the time till the coalesced droplet reaches its maximum expansion, is not affected by the addition of polyelectrolytes since the contact line does not encounter any surface defects in the expansion phase.

The droplet behaves differently in the retraction phase. Figure 3d shows that increasing the polyelectrolyte concentration increases the contact time, which becomes infinity for high concentrations, as the droplet sticks to the surface. It has been shown earlier that the retraction rate 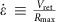 for single drop impacts is a material property and does not depend on the impact velocity^29^,^46^. We plot the retraction rate in Supplementary Fig. 4 for drop-on-drop impacts with various drop sizes, impact velocities and polyelectrolyte concentrations. We observe that the retraction rate does not depend on the impact velocity, similar to the case of single drop impacts^29^,^46^, and decreases with polyelectrolyte concentration.

The decrease in retraction rate arises from the pinning of the contact line on the defects formed by polyelectrolyte precipitation. The contact line may get pinned and depinned several times during the retraction phase, and each of these events dissipates some energy and slows the retraction. The final outcome of the impact depends on how much energy is dissipated by pinning and depinning. We observe that for low concentrations of polyelectrolytes, bouncing might still occur, while for higher concentrations droplets are arrested. Defect density, and thus the ability to arrest impacting droplets, intuitively increases with concentration. The objective is then to find the minimum concentration for which the number of generated defects arrests the impacting droplets on the surface. To determine this minimum concentration, we consider the energy balance during impact. Viscous effects are weak here as the Ohnesorge number^47^ 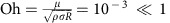. An impacting droplet striking a non-wetting surface will rebound if enough of its initial kinetic energy, which is converted into surface energy during the expansion phase, is recovered during the retracting phase^27^. Since viscous dissipation is negligible, the only mechanism for the droplet to lose energy is through the pinning forces on the surface. However, part of the initial kinetic energy is also converted into internal vibration energy due to droplet oscillations^48^,^49^,^50^. We estimate the fraction of the initial kinetic energy that gets converted into vibration energy as (1−*e*_0_^2^)*E*_k_ where *e*_0_ is the restitution coefficient for the base case of drop-on-drop impacts of pure water droplets, where there is negligible pinning^51^. The restitution coefficient is calculated as 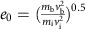, where *m*_i_,*m*_b_ are the masses of the droplet and *v*_i_,*v*_b_ are the velocities of the center of mass before and after impact (here *m*_b_/*m*_i_=2). As shown in Fig. 3e, despite some scatter in the data, we find that the restitution coefficient of pure water droplets does not strongly depend on the Weber number of the impacting droplet and is on the order of 0.4 for our impact experiments. Assuming that the internal vibration of the droplets is not significantly affected by polyelectrolytes, this measured restitution coefficient for water droplets can be used to estimate the vibration energy of polyelectrolyte droplets. Hence, polyelectrolyte droplets will stick to the surface if the work of pinning during the retraction phase exceeds the remaining kinetic energy *e*_0_^2^*E*_k_.

In quantitative terms, the initial kinetic energy of the droplet is *E*_k_∼*ρR*^3^*V*^2^, and the work of pinning on one single defect is *W*∼*σl*^2^, where *R* is the radius of the droplet, *V* is its impact velocity, *ρ* is the density, *σ* is the surface tension and *l* is the defect size^52^,^53^,^54^. The total work of pinning during the receding phase is then *W*∼*σl*^2^ *N*_defects_, where *N*_defects_ is the total number of defects under the droplet during the impact. We can then define an average surface concentration of defects *C*_s_, and *W*∼*σl*^2^ *R*^2^*C*_s_. The ratio of kinetic energy and the work of pinning gives the characteristic non-dimensional number for this problem, the pinning number 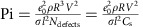, which should govern the transition between bouncing and sticking.

The surface concentration of defects is hard to estimate, and can vary from zero to a maximal value corresponding to the case where all the polyelectrolytes in both droplets react and form surface precipitates. In an attempt to have an expression of Pi as a function of the controlled parameters of the problem, we make the following hypothesis: the number of surface defects is a fraction of the total number of defects that can be created by all the polyelectrolytes in the droplet 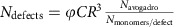, where *C* is defined as the volumetric molar concentration of monomers and *ϕ* is the fraction of polyelectrolytes that precipitated during the impact, which can be estimated, for short contact times, as the ratio of the contact time to the precipitation time. The pinning number is then


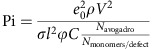


To estimate Pi, we use SEM images to estimate the average defect size *l*, which was around 500 nm. To estimate the number of monomers per defect, we relate the radius of a semi-flexible polymer chain to the number of its monomers, using the standard Flory theory, which gives *l*∼*aN*^*υ*^, where *l* is the defect size and *a* is the monomer size (∼1 nm) (ref. 55). In the case of polyelectrolytes, *υ*=(3)/(5), and this leads to a number *N* on the order 10,000 monomers per defect.

To determine the ratio *ϕ*, we compare the precipitation time to the contact time. As diffusion effects are negligible during contact time (*τ*_diffusion_≫*τ*_contact_), in our experiments, precipitation is mainly driven by mixing of the droplets. When the two droplets coalesce, they need some time to completely mix, as shown schematically in Supplementary Fig. 5a. During the mixing phase, only a fraction of the polyelectrolyte molecules have come into contact with each other and are able to interact. The mixing is driven by inertial and capillary forces and the mixing time scales as 
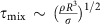
 (refs 56, 57). The precipitation rate then scales as 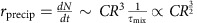. We experimentally measured the precipitation rate of two coalescing droplets with opposite polyelectrolytes, which was indeed found to scale as 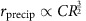 as predicted (See Supplementary Note 1 and Supplementary Fig. 5 for details). The contact time has the same scaling law as the mixing time 
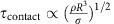
, but with a different pre-factor^27^. Thus, we expect *ϕ* to be independent of the drop parameters and only depend on the ratio of the pre-factors. The experimentally measured pre-factor is larger for the mixing time than for the contact time by about an order of magnitude, so 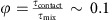.

**Figure 4:**
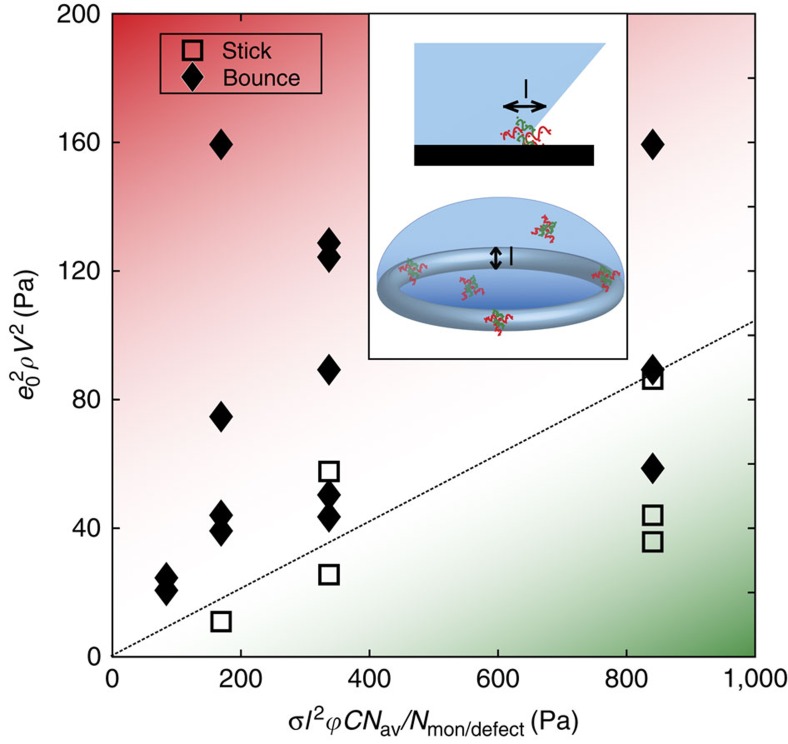
Bouncing-Sticking transition in a two-drop impact. The data points are experimental outcomes of impacts at different concentrations, radii and impact velocities. The figure axes are the work of pinning and the kinetic energy of the droplet. The dashed line roughly indicates the transition between bouncing and sticking and corresponds to Pi∼0.1. Below the dashed line, pinning forces are larger than inertia and pinning of the droplet is expected, while bouncing is expected above. The inset illustrates the defect size, and the region around the contact line that is acted upon by pinning forces at a certain time.

We performed several drop-on-drop impact experiments, varying the concentration, radius and velocity, and recorded whether the outcome was bouncing or sticking. In Fig. 4, we can see that for high kinetic energies, or high Pi, droplets bounce, while they tend to stick for lower Pi. The transition occurs at a pinning number of around 0.1. Our model of Pi remains a first-order attempt to capture the physics of the phenomenon, and more detailed studies on the coupling between precipitation and impact dynamics and their effect on the size and density of defects are needed to refine the model.

Finally, the retention of sprayed liquids was measured for different surfaces and liquids. Sprayers were used to deliver fixed amounts of liquid to the surface in the form of jets of fine droplets, at constant jet velocity and cone angle. Two metrics were used to quantify the retention. First, the mass of the liquid retained by the sample was measured by weighing the sample after every spray^58^. Second, we measured the surface coverage, i.e., the area covered by the liquid divided by the total area of the substrate^59^. The first metric characterizes how much liquid can stick to the surface, while the second metric quantifies the uniformity of the coverage.

**Figure 5:**
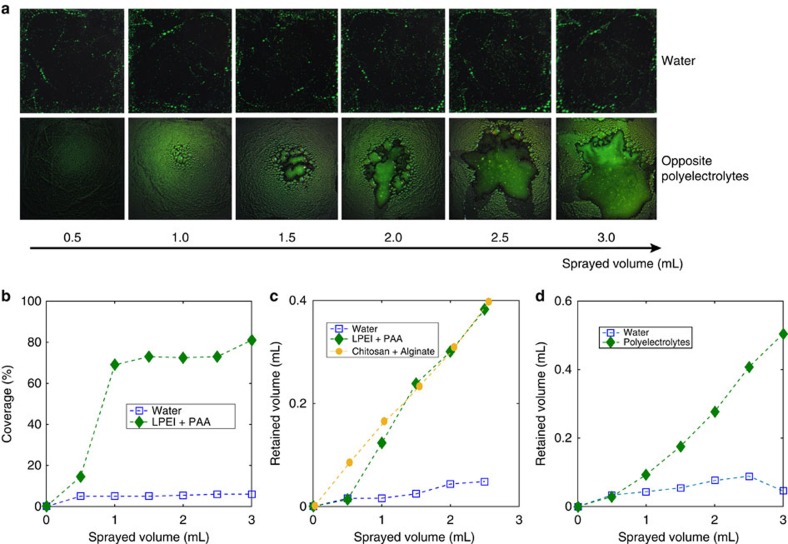
Water and opposite polyelectrolytes spraying on a superhydrophobic surface. (**a**) Photographs of a 2 × 2” superhydrophobic surface after spraying fixed volumes of water and polyelectrolytes (linear polyethyleneimine (LPEI) and polyacrylic acid (PAA)). A fluorescent dye was added to allow visualization. (**b**) Coverage of the surface by the liquid in the same experiments. (**c**) Retained volume of liquid on the same surface after spraying water, LPEI+PAA and Chitosan + Alginate. (**d**) Retained volume of Chitosan + Alginate on a lotus leaf.

Figure 5a shows images of the surfaces (2 × 2 inches), as different liquids are sprayed. A fluorescent dye was added to all the sprayed liquids for better visualization. As expected, for a superhydrophobic surface, very little water was retained and the coverage did not exceed 7% after 3 ml were sprayed, as shown in Fig. 5b. However, when oppositely charged polyelectrolytes (concentration 20 mM) were simultaneously sprayed on the superhydrophobic surface, much more of the liquid remained on the surface: the coverage was 70% after 1 ml and reached 80% when 3 ml of liquid were sprayed. The retained volume exhibits similar trends, as shown in Fig. 5c. When polyelectrolytes are sprayed, the retained volume continuously increases, and it only stops when the sample eventually cannot hold more liquid and further spraying removes the excess accumulated liquid. There is a 10-fold increase in retention as compared with water and the retained volume is even close to the retained volume of water on a superhydrophilic surface that we measured in separate experiments (Supplementary Fig. 6), and which provides an estimate of the maximal attainable value for retention.

Spray experiments with a polyelectrolyte concentration of 2 mM resulted in zero retention while experiments with 20 mM showed large enhancements in retention. These results are consistent with our Pi model, which predicts the transition from bouncing to sticking drops to occur at a concentration of ∼10 mM for our sprays.

We also show similar retention properties using different polyelectrolyte molecules. Retained volume using Chitosan (positively charged) and Alginate (negatively charged) is also largely increased, as shown in Fig. 5c. These polyelectrolytes are polysaccharides that are non-toxic, biocompatible and biodegradable, which makes them excellent candidates for plant treatment^60^,^61^,^62^. We finally sprayed these polyelectrolytes on superhydrophobic lotus leaves and observed a similar pattern of increased retention (Fig. 5d), showing their efficiency on natural surfaces as well. The method described here is not limited to superhydrophobic surfaces and works similarly on hydrophobic surfaces (Supplementary Fig. 7), which makes it applicable to a large variety of plants and surfaces in practice.

We demonstrate here a new mechanism to enhance spray deposition on hydrophobic surfaces through *in-situ* precipitation of polyelectrolytes on the surface. We show how defects formed *in-situ* on the surface during the impact can pin the impinging droplets, and show the advantages of creating these hydrophilic wetting defects *in-situ*. We study the mechanism of precipitate formation in coalescing droplets, which gives insights on the extent of the precipitation reaction during droplet impacts, and we propose a simple model balancing kinetic and pinning energies that leads to a criterion characterizing the transition between bouncing and sticking droplets or sprays. This method allows surface modification and deposition of the liquid of interest in one single step and introduces a new control parameter in the design space for sprays, which is the polyelectrolyte concentration. We show that this method could work on different surfaces and with different types of polyelectrolytes, as long as their zeta potential is high enough to interact, and there are several natural, biodegradable and readily available polyelectrolytes that can be used. It is known that low retention of pesticides on hydrophobic plants is a major problem in agriculture^6^. By adding small amounts of these polyelectrolytes to sprays, the quantity of pesticides used could be significantly reduced, while coverage is increased, offering full protection to the plant and limiting the toxic effects of pesticides. This method can also be used for other agricultural sprays, paints, and any other process that involves sprays or droplet deposition.

## Methods

### Fabrication of superhydrophobic surfaces

Plasma etch with O_2_ and SF_6_ was performed on silicon substrates to make a silicon nanograss texture. The latter is a superhydrophilic surface with contact angles around 0°. To make it superhydrophobic, the surface was coated with a hydrophobic modifier (octadecyltrichlorosilane). Advancing and receding contact angles of deionized water on a nanograss silicon surface treated with the hydrophobic modifier were measured with a goniometer (Model 500, ramé-hart) at 25 °C to be 165°±2° and 160°±3°, respectively.

### Polyelectrolyte solutions

Four polyelectrolyte molecules were used. All the polyelectrolytes were obtained from Sigma- Aldrich and used as received. The properties of the used solutions are in Table 1.

The pH was adjusted using HCl and NaOH for all solutions except the one with chitosan that was adjusted by adding acetic acid.

### Spraying method

An airbrush was vertically fixed, 21 cm above the horizontal sample. The pressure of the air supply to the airbrush was maintained constant throughout the whole set of experiments so as to keep the same jet velocity and cone angle. The liquid was delivered to the airbrush by inputs of 500 μl using a syringe. The samples (2′′−2′′) were chosen to be bigger than the spray cone (more than 98% of the sprayed liquid hits the sample).

### Coverage determination

A small quantity of fluorescent dye (Fluorescein sodium salt) was added to the sprayed solutions. Imaging under ultraviolet light was realized after each spray, and image processing using ImageJ was performed to determine the fraction of the surface covered by the liquid.

### Data availability

The authors declare that the data supporting the findings of this study are available within the article and its supplementary information.

## Additional information

**How to cite this article**: Damak, M. *et al*. Enhancing droplet deposition through *in-situ* precipitation. *Nat. Commun.* 7:12560 doi: 10.1038/ncomms12560 (2016).

## Supplementary Material

Supplementary Information Supplementary Figures 1-7 and Supplementary Note 1

Supplementary Movie 1 Water spraying on a superhydrophobic surface.

Supplementary Movie 2 Simultaneous spraying of LPEI and PAA on a superhydrophobic surface.

Supplementary Movie 3 Side view of an impact of a droplet containing PAA on a droplet containing LPEI deposited on a superhydrophobic surface.

Supplementary Movie 4 Top view of an impact of a droplet containing PAA on a droplet containing LPEI deposited on a superhydrophobic surface.

Supplementary Movie 5 Precipitate formation upon coalescence of two droplets containing LPEI and PAA respectively and held in the air by needles.

## Figures and Tables

**Table 1:** Solutions properties.

**Polyelectrolyte**	**Molar weight (kg** **mol**^−**1**^)	**Concentration (mM)**	**pH**	**Zeta potential (mV)**
Linear polyethyleneimine (LPEI)	20	2, 5, 10, 20, 50	4.3–4.7	43
Polyacrylic acid (PAA)	20	2, 5, 10, 20, 50	4.3–4.7	−45
Chitosan	60	10	4.4	52.3
Alginate	60	10	3.1	−33.7

Molar weight, concentration, pH and zeta potential of the polyelectrolyte solutions used in the experiments.
