# Author Correction: Enhancing droplet deposition through in-situ precipitation

**DOI:** 10.1038/s41467-020-18179-0

**Published:** 2020-08-21

**Authors:** Maher Damak, Md Nasim Hyder, Kripa K. Varanasi

**Affiliations:** Department of Mechanical Engineering, Massachusetts Institute of Technology, 77 Massachusetts avenue, Cambridge, MA 02139 USA

Correction to: *Nature Communications* 10.1038/ncomms12560, published online 30 August 2016.

The original version of this article included Seyed Reza Mahmoudi, who was from “Department of Mechanical Engineering, Massachusetts Institute of Technology, 77 Massachusetts avenue, Cambridge, 02139, Massachusetts, USA”, as the second author in the author list. Subsequently this person stated that his contribution to the work was not sufficient to be included as an author. Consequently, this name has been removed from the author list. In addition, the sentence “M.D., S.R.D, N.H. and K.K.V. conceived the project” in the Author Contributions section has been corrected to “M.D., N.H. and K.K.V. conceived the project”. This has been corrected in both the PDF and HTML versions of the article.

