# Supplementary material for: Enhancing droplet deposition through in-situ precipitation
Source: Nat Commun. 2016 Aug 30;7:12560. doi: 10.1038/ncomms12560 (PMC5013560; doi:10.1038/ncomms12560)
Supplement: Supplementary Information — Supplementary Figures 1-7 and Supplementary Note 1 [file ncomms12560-s1.pdf]

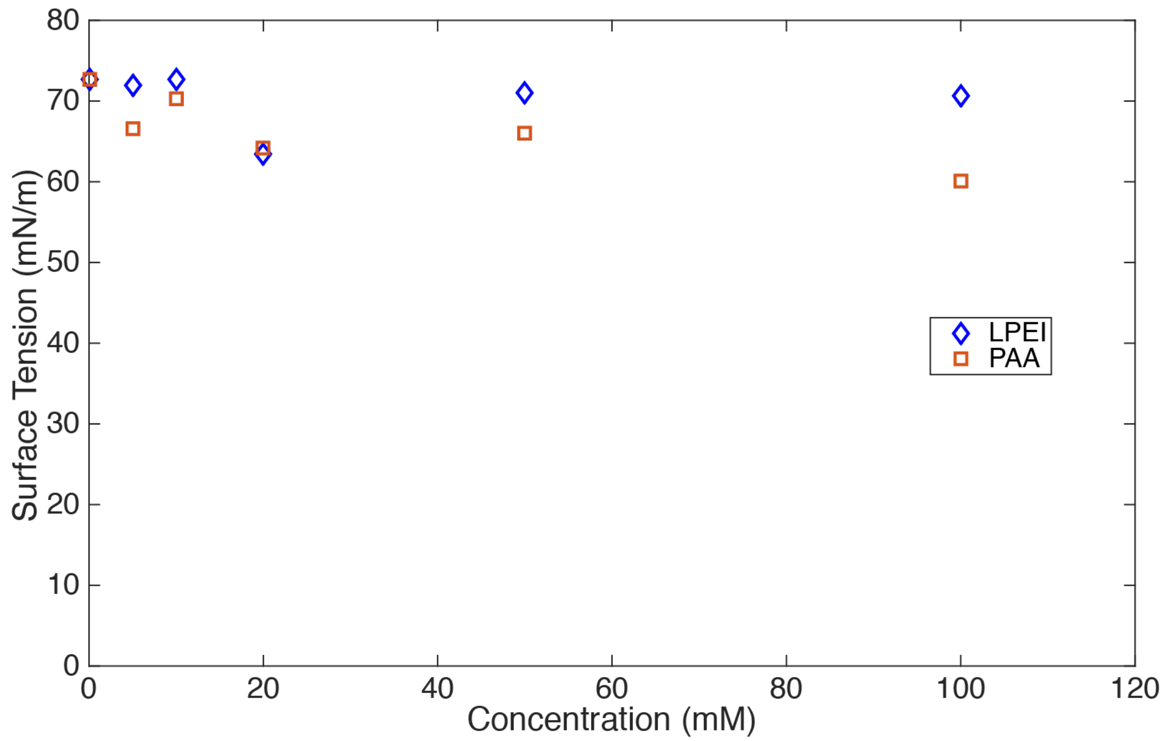

Supplementary Figure 1 | Surface tension of polyelectrolyte solutions. Experimentally measured values of surface tension of the solutions that were used in experiments throughout the paper. All surface tensions of used solutions remained between 63 and 73mN/m. The changes in surface tension did not seem to be attributed to the polyelectrolytes but rather to the addition of NaOH and HCl to the solutions.

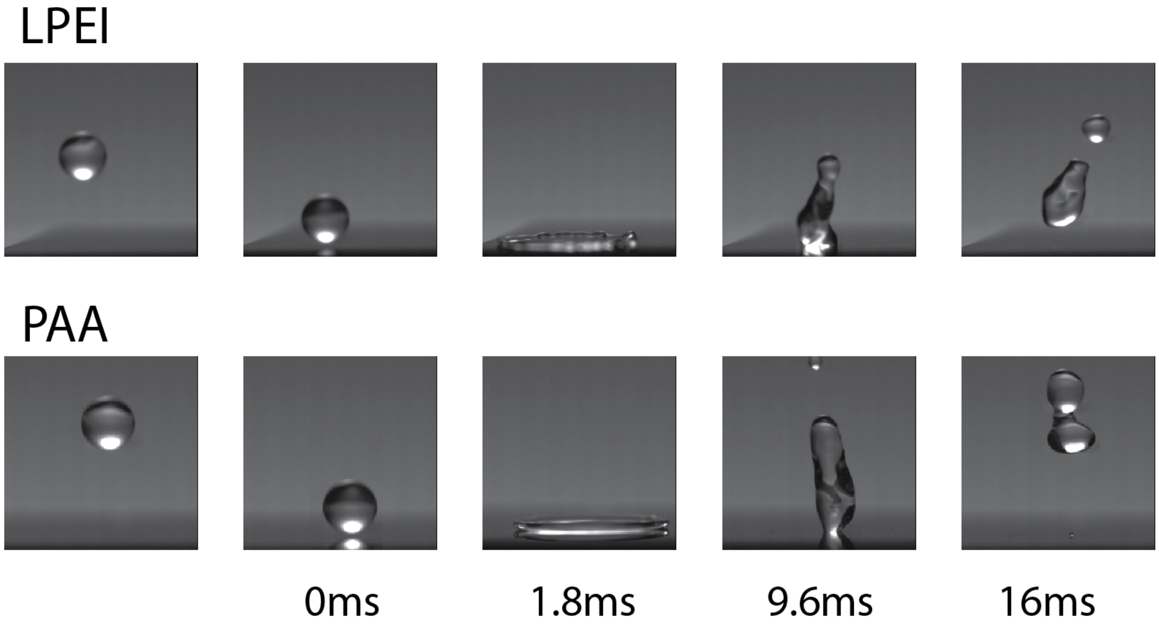

Supplementary Figure 2 | Impact of positive and negative polyelectrolyte droplets on a superhydrophobic surface. Both LPEI and PAA droplets bounce off the surface. The presence of a single polyelectrolyte does not seem to influence the impact behavior.

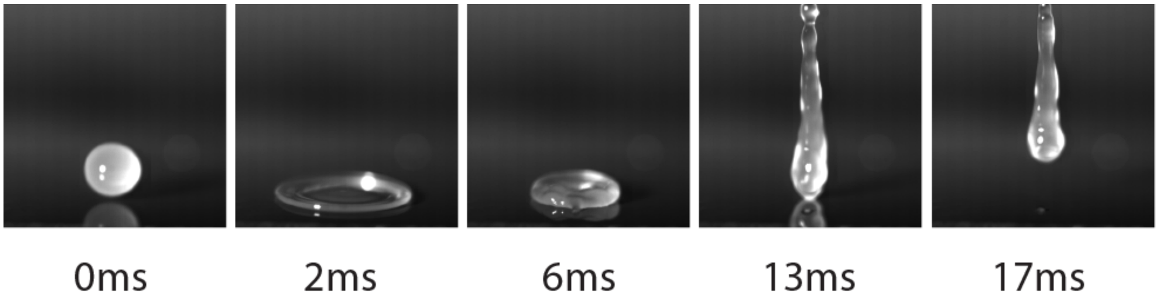

Supplementary Figure 3 | Impact of a water droplet containing 3µm silica particles on a superhydrophobic surface. The droplet bounces off in a similar fashion to a pure water droplet. No silica particles are left on the surface.

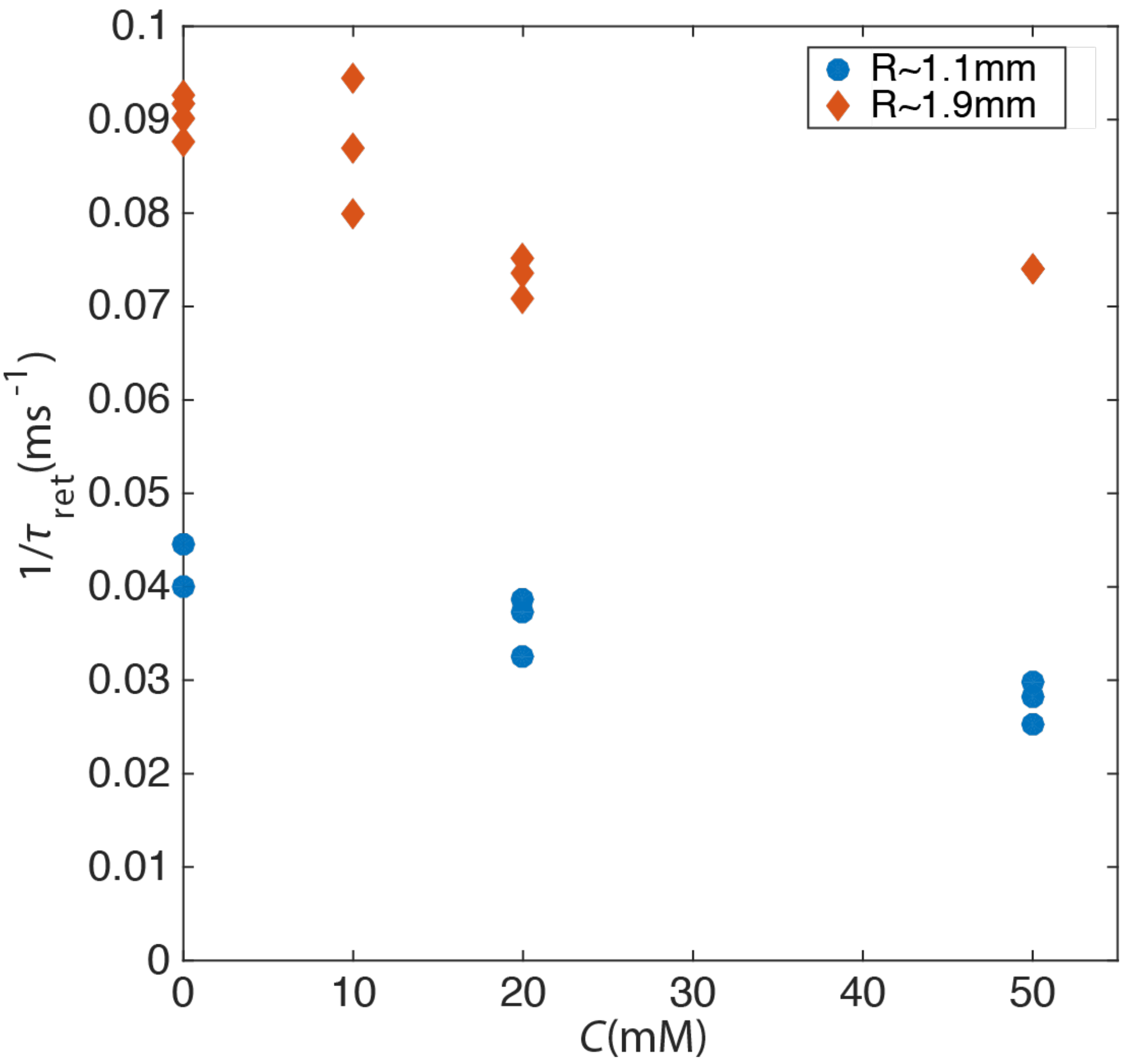

Supplementary Figure 4 | Retraction rate in drop-on-drop experiments as a function of polyelectrolyte concentration for two droplet sizes and different impact velocities. In the case of bouncing droplets the retraction rate is the inverse of the retraction time, defined as the time from reaching the maximum spreading to bouncing. $\dot{\varepsilon} \sim \frac{1}{\tau_{\mathrm{ret}}}$.

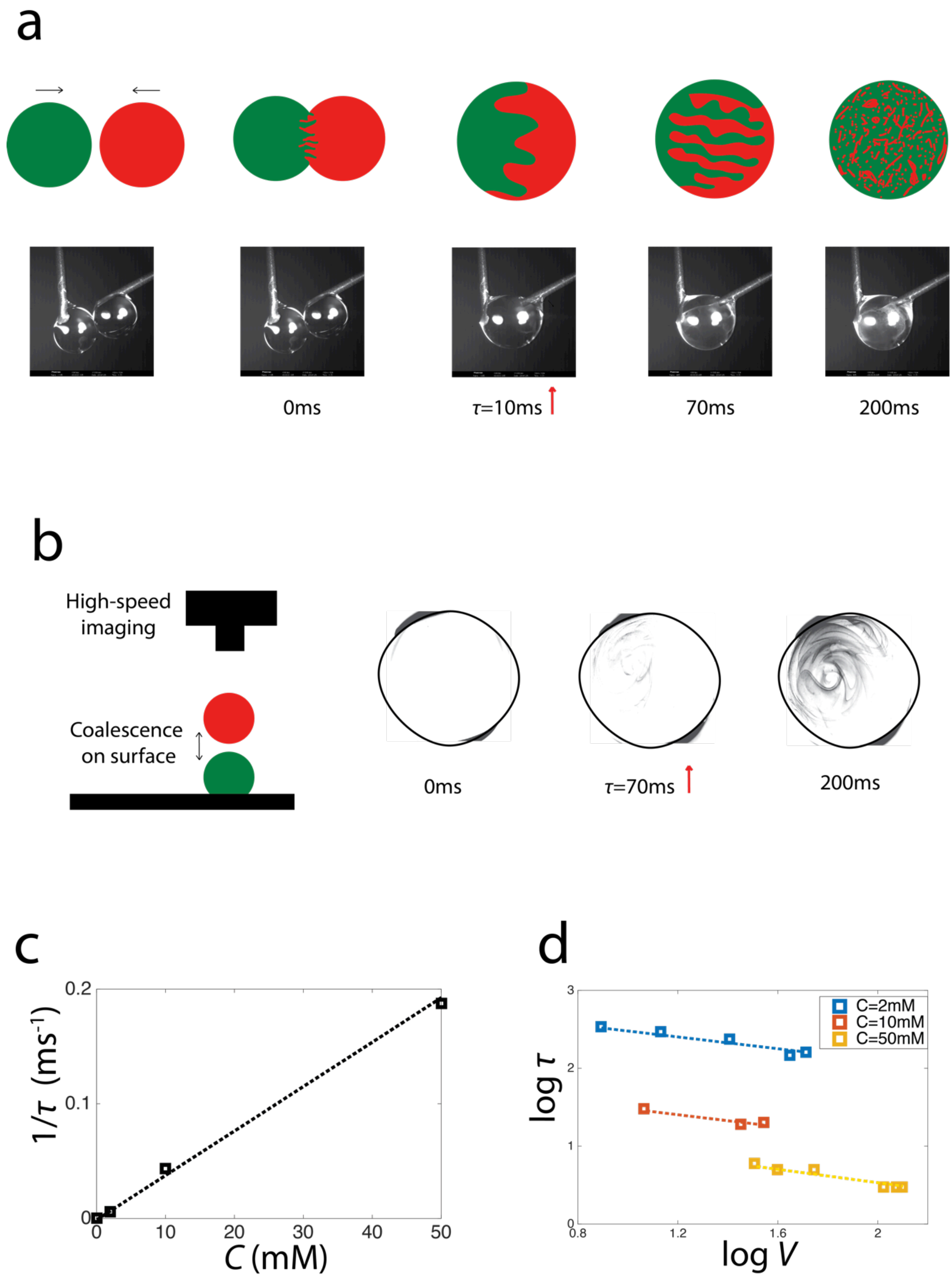

Supplementary Figure 5 | Precipitate formation upon coalescence of two droplets.
(a) Schematics and snapshots of the mixing of two polyelectrolyte droplets and the

precipitate formation in air. For complete movie, see Supplementary Movie 5. **(b)** Schematic of experiments and snapshots of droplet mixing on a hydrophobized glass surface. The images were treated for better visualization. The snapshots corresponding to the precipitate formation time τ are indicated in (a) and (b). **(c)** Experimental precipitation time variation with polyelectrolyte concentration **(d)** log-log graph of the precipitation time (in ms) as a function of the volume of the droplets (in mm^3). The fitting lines have a slope of -1/2.

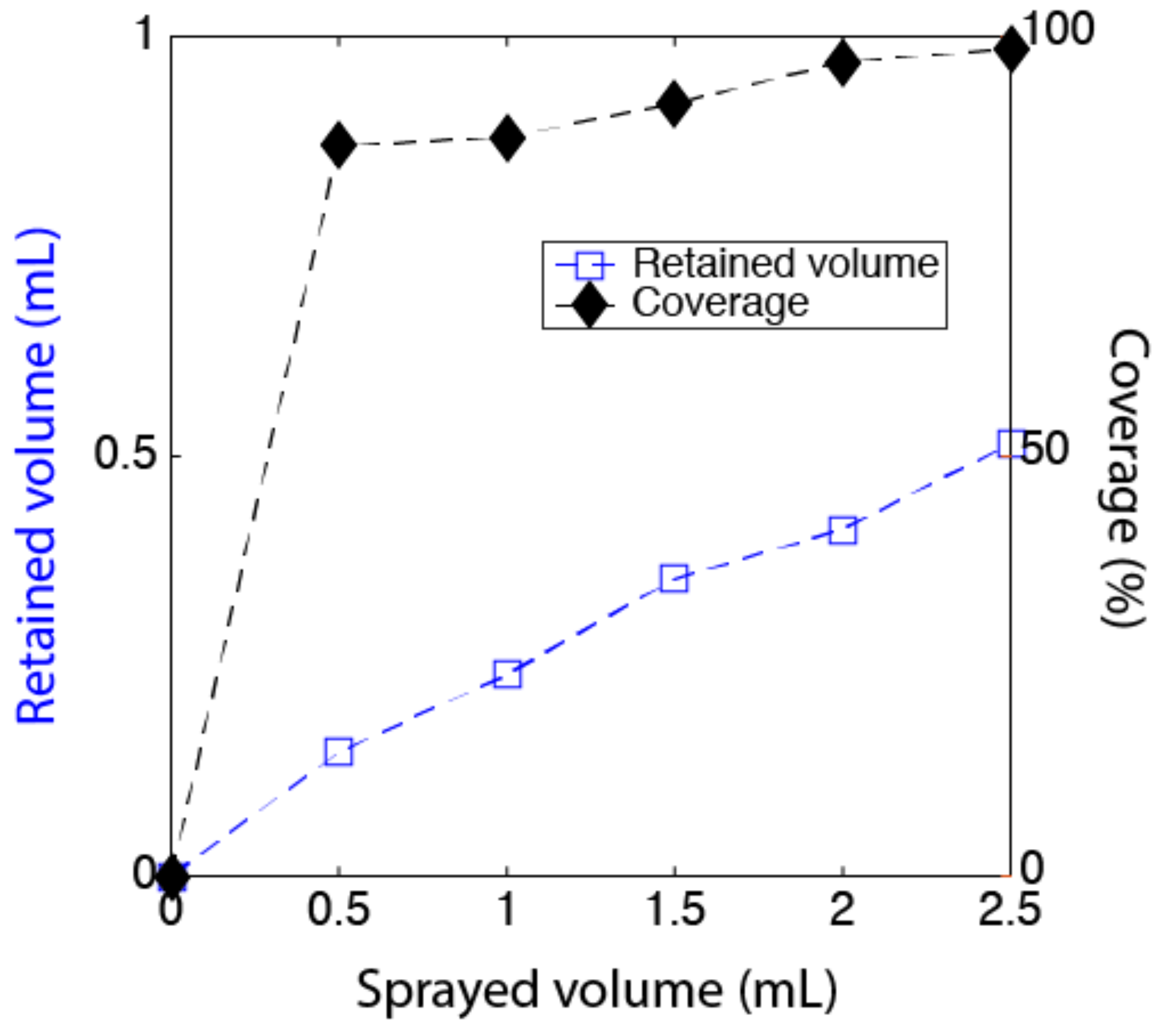

Supplementary Figure 6 | Retention and coverage after spraying water on a superhydrophilic surface. The surface was silicon nanograss, with a contact angle close to 0˚. The values of retained volume (left axis) and surface coverage (right axis) are slightly larger than those for polyelectrolyte spraying on superhydrophobic surfaces.

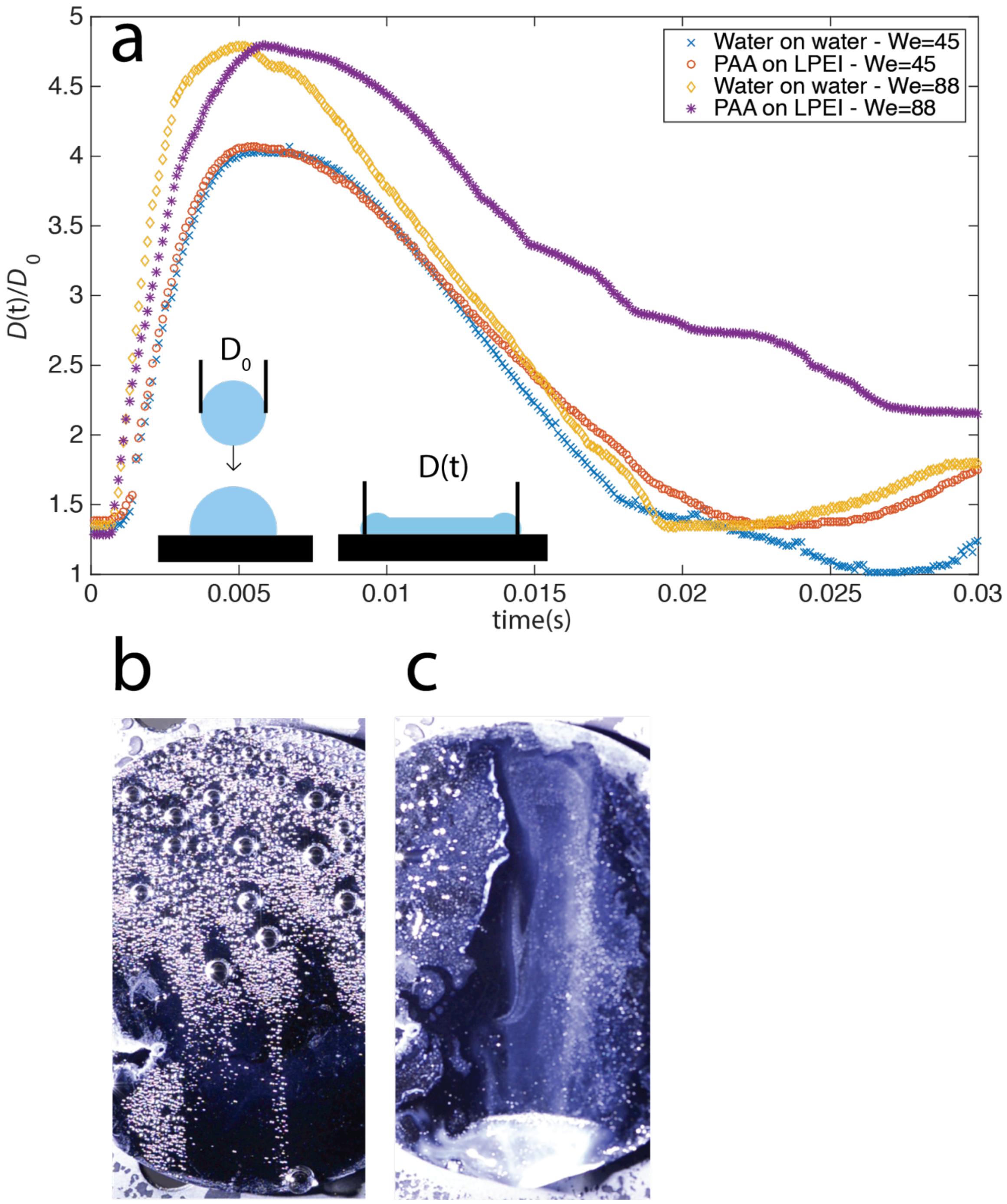

Supplementary Figure 7 | Drop-on-drop impacts and sprays on hydrophobic smooth OTS-coated silicon surface. Advancing and receding angles are 110˚ and 96˚ respectively. No bouncing of single droplets is observed for any Weber number. **(a)** Normalized contact length as a function of time for four drop-on-drop impacts (Water and polyelectrolytes for two different Weber numbers). The droplet radius was 1.1mm for all experiments. Adding polyelectrolytes does not affect the maximum spreading

diameter but slows down the retraction in the two Weber number regimes considered. Polyelectrolyte solutions also retract to a minimum diameter that is higher than water. **(b-c)** Snapshots after spraying a similar quantity of liquid on a vertically oriented surface. Water was sprayed in (b) and LPEI and PAA were simultaneously sprayed in (c). In the case of water, individual droplets could be seen, and, as soon as they became big enough, droplets shed, entraining other droplets in their wake and leaving substantial parts of the surface uncovered. In (c), polyelectrolyte spraying leads to a uniform film that covers most of the surface.

Supplementary note 1 – Measurement of the rate of precipitation

To measure the rate of precipitation, we filmed the coalescence of two droplets containing oppositely charged polyelectrolytes, both in air and on a surface, using a high-speed camera. Supplementary Fig. 4a shows side-view snapshots of the precipitate formation in coalescing droplets in air, and Supplementary Fig. 4b shows top-view snapshots of the coalescence on a hydrophobic glass surface (Contact angle 107° ± 3°). In both cases, whitish precipitates appear as filaments and grow inside the droplet. We measured, for different drop sizes and concentrations, the time τ_{exp} at which the precipitates become large enough to be resolved by our camera (when their thickness is ~ 20µm) and estimated to first order the rate of the precipitation reaction r_{precip}, as

$$r_{\mathrm{precip}} = \frac{dN}{dt} \propto \frac{1}{\tau_{\mathrm{exp}}} \quad (1)$$

where *N* is the number of defects formed.

While τ_{exp} depends on the imaging conditions, relative values of τ_{exp} can be used to compare precipitation rates under identical imaging conditions when the concentration and droplet volume are varied. Each experiment was repeated several times, and consistent values of τ_{exp} were obtained. The values of τ_{exp} ranged in the tens of milliseconds, the contact time being around 15ms. The results are shown in Supplementary Fig. 4c and 4d. We find that the precipitation time is inversely proportional to the polyelectrolyte concentration, and, while the droplet size could not be varied over a large range, we can see a trend of the precipitation time varying as the volume of the droplets to the power -0.5. Thus, the scaling law for the precipitation rate is $r_{\mathrm{precip}} \propto CV^{\frac{1}{2}} \propto CR^{\frac{3}{2}}$.
